# Serotonin-specific neurons differentiated from human iPSCs form distinct subtypes with synaptic protein assembly

**DOI:** 10.1007/s00702-021-02303-5

**Published:** 2021-02-09

**Authors:** Charline Jansch, Georg C. Ziegler, Andrea Forero, Sina Gredy, Sina Wäldchen, Maria Rosaria Vitale, Evgeniy Svirin, Johanna E. M. Zöller, Jonas Waider, Katharina Günther, Frank Edenhofer, Markus Sauer, Erhard Wischmeyer, Klaus-Peter Lesch

**Affiliations:** 1 Division of Molecular Psychiatry, Center of Mental Health, University of Würzburg, Margarete-Höppel-Platz 1, 97080 Würzburg, Germany; 2 Department of Psychiatry, Psychosomatics and Psychotherapy, Center of Mental Health, University of Würzburg, Würzburg, Germany; 3 Institute of Physiology, Molecular Electrophysiology, University of Würzburg, Würzburg, Germany; 4 Laboratory of Psychiatric Neurobiology, Institute of Molecular Medicine, Sechenov First Moscow State Medical University, Moscow, Russia; 5 Department of Biotechnology and Biophysics, Biocenter, University of Würzburg, Würzburg, Germany; 6 Department of Translational Neuroscience, School for Mental Health and Neuroscience (MHeNS), Maastricht University, Maastricht, The Netherlands; 7 Department of Genomics, Stem Cell Biology and Regenerative Medicine, Institute of Molecular Biology and CMBI, Leopold-Franzens-University Innsbruck, Innsbruck, Austria; 8 Institute of Molecular Regenerative Medicine, SCI-TReCS, Paracelsus Medical University, Salzburg, Austria

**Keywords:** Human induced pluripotent stem cell (hiPSC), Serotonin-specific neurons, Median and dorsal raphe, Synapse formation, Cadherin-13 (CDH13), Neuropsychiatric disorders

## Abstract

Human induced pluripotent stem cells (hiPSCs) have revolutionized the generation of experimental disease models, but the development of protocols for the differentiation of functionally active neuronal subtypes with defined specification is still in its infancy. While dysfunction of the brain serotonin (5-HT) system has been implicated in the etiology of various neuropsychiatric disorders, investigation of functional human 5-HT specific neurons in vitro has been restricted by technical limitations. We describe an efficient generation of functionally active neurons from hiPSCs displaying 5-HT specification by modification of a previously reported protocol. Furthermore, 5-HT specific neurons were characterized using high-end fluorescence imaging including super-resolution microscopy in combination with electrophysiological techniques. Differentiated hiPSCs synthesize 5-HT, express specific markers, such as tryptophan hydroxylase 2 and 5-HT transporter, and exhibit an electrophysiological signature characteristic of serotonergic neurons, with spontaneous rhythmic activities, broad action potentials and large afterhyperpolarization potentials. 5-HT specific neurons form synapses reflected by the expression of pre- and postsynaptic proteins, such as Bassoon and Homer. The distribution pattern of Bassoon, a marker of the active zone along the soma and extensions of neurons, indicates functionality via volume transmission. Among the high percentage of 5-HT specific neurons (~ 42%), a subpopulation of CDH13 + cells presumably designates dorsal raphe neurons. hiPSC-derived 5-HT specific neuronal cell cultures reflect the heterogeneous nature of dorsal and median raphe nuclei and may facilitate examining the association of serotonergic neuron subpopulations with neuropsychiatric disorders.

**Supplementary Information:**

The online version contains supplementary material available at 10.1007/s00702-021-02303-5.

## Introduction

A complex interplay of multiple genes and environmental factors causes abnormalities in brain development increasing the risk for neurodevelopmental and psychiatric disorders (Lesch et al. 2011; Wang et al. 2009). These diseases are associated with alterations at both structural and functional brain levels implicating, among others, monoaminergic systems. Accordingly, dysregulation of serotonin (5-HT), norepinephrine, and dopamine signaling in the brain have been linked to the pathogenesis of affective and psychotic disorders (Ashok et al. 2017; Lissemore et al. 2018; Siuta et al. 2010). 5-HT is released throughout the entire central nervous system (CNS) and exerts its action by modulation of sensory processing, cognitive control, and emotion regulation. Furthermore, 5-HT regulates developmental processes, such as cell proliferation, migration, differentiation, maturation, and survival (Lesch and Waider 2012). Different 5-HT receptors promote neural expansion and facilitate neuronal interactions (Banasr et al. 2003; Zhang 2003). Most of the studies published on the biophysical characteristics of the 5-HT system were conducted in animal models (Gutknecht et al. 2008; Jones et al. 2015; Norton et al. 2008; Saigal et al. 2013; Waider et al. 2017). The molecular and cellular intricacies of 5-HT signaling in humans and their impact on health and disease, however, are still largely unknown (for review, Daubert and Condron 2010; Erzurumlu and Gaspar 2012; Garbarino et al. 2019; Gaspar et al. 2003; Lesch and Waider 2012; Marazziti 2017).

The brain 5-HT system originates from the raphe nuclei. These nuclei extend along the midline of the brainstem and are separated into a rostral group, responsible for the widespread innervation of numerous brain regions, and a caudal group, which projects to the spinal cord (Alonso et al. 2013; Kiyasova and Gaspar 2011; Muzerelle et al. 2016). During development, ventral rostral hindbrain progenitors arise from rhombomeric (r) segments 2–3 and generate median raphe 5-HT specific neurons (Alonso et al. 2013; Bang et al. 2012), whereas the dorsal raphe nucleus arises from rhombomere 1 (Jensen et al. 2008). The allocation of 5-HT specific neurons to a median and dorsal raphe origin is of clinical interest as the two raphe nuclei play differential roles in social and addictive behavior (Balazsfi et al. 2018; Verheij et al. 2018).

Cadherin-13 (CDH13) is implicated in multiple neurodevelopmental disorders, such as autism spectrum disorders (Sanders et al. 2011, 2015), attention-deficit/hyperactivity disorder (ADHD) (Lasky-Su et al. 2008; Lesch et al. 2008; Neale et al. 2010), major depressive disorder (Edwards et al. 2012), and substance use disorders (Hart et al. 2012; Treutlein et al. 2009; Uhl et al. 2008). As a negative regulator of axonal outgrowth (Fredette et al. 1996) CDH13 contributes to the development of the raphe nuclei. During the development of the mouse 5-HT system, CDH13 is enriched in the lateral wing of the dorsal raphe nucleus, whereas the median raphe is spared. CDH13 influences cell density and guides outgrowth of serotonergic neurons to the prefrontal cortex thereby influencing impulsive behavior as well as learning and memory (Forero et al. 2020; Forero et al. 2017).

The reprogramming of human adult fibroblasts into pluripotent stem cells by ectopic expression of four transcription factors revolutionized stem cell research both in terms of ethical concerns and routine applicability (Ardhanareeswaran et al. 2017; Brennand et al. 2012; Kaiser and Feng 2015). Human induced pluripotent stem cells (hiPSCs) are now a widely employed tool to generate patient-derived cell, tissue, and organoid models with the potential to reveal molecular mechanisms underlying the pathogenesis of neurodevelopmental disorders (Brennand et al. 2011; Halevy et al. 2015; Mariani et al. 2015). The successful generation of patient-derived disease models for neurodevelopmental disorders depends on reliable and robust methods for the differentiation of neuronal subtypes from hiPSCs. This field of research requires replication and refinement of previously established differentiation approaches (McNeill et al. 2020). Several protocols describe the generation of specific neuronal subtypes, such as motor neurons (Intoh et al. 2016), dopamine (Fedele et al. 2017; Kirkeby et al. 2017; Kriks et al. 2011; Sanchez-Danes et al. 2012; Suzuki et al. 2017; Tofoli et al. 2019), glutamate (Cao et al. 2017; Gunhanlar et al. 2018; Shi et al. 2012), and γ-aminobutyric acid (GABA)-specific (Merkle et al. 2015; Yang et al. 2017) neurons while relatively little is known about the differentiation into serotonergic neurons (Lu et al. 2016; Vadodaria et al. 2016).

A previously reported method for the generation of 5-HT specific neurons from hiPSCs provides mainly median raphe 5-HT specific neurons (Lu et al. 2016). The protocol relies on the addition of small molecules which initiate a time- and concentration-dependent recapitulation of neurodevelopmental pathways by patterning hindbrain development (Lu et al. 2016). Here, we aimed to replicate this hindbrain differentiation method for the generation of 5-HT specific neurons from hiPSCs to provide an in-depth characterization of these cells. We validated the successful differentiation of serotonergic neurons by specific markers including tryptophan hydroxylase 2 (TPH2), 5-HT transporter (5-HTT), and 5-HT. Additionally, we assessed functionality of 5-HT specific neurons by electrophysiological recordings in the longitudinal course over 6 weeks. Finally, we identified subpopulations of 5-HT specific neurons by immunocytochemical analyses of CDH13 expression and examined their ability to form interneuronal connections by super-resolution imaging of synaptic markers.

## Material and methods

### Reprogramming of human fibroblasts into hiPSCs

The hiPSC line UKWMPi001-B was generated by reprogramming fibroblasts of a healthy donor using the CytoTune-iPS Reprogramming Kit 2.0 (Thermo Fisher Scientific, Waltham, MA, USA) as previously described (Jansch et al. 2018). For a detailed description of the reprogramming procedure, see SM. A further hiPSC line JMUi001-A (Kwok et al. 2018) reprogrammed by a lentiviral vector (Somers et al. 2010) from normal human dermal fibroblasts (NHDFs, Promocell) was used to verify the establishment of the differentiation procedure in terms of the expression of 5-HT specific markers and CDH13, as well as the electrophysiological signature of the generated 5-HT specific neurons (data not shown). Pluripotent capacity of both hiPSC lines was assessed by immunostaining (OCT3, SSEA-4, TRA-1-60) and FACS (TRA-1-60) or qRT-PCR (*NANOG*, *OCT3*, *REX1*) of pluripotency factors, and by spontaneous differentiation into all three germ layers after embryoid body (EB) formation. Chromosomal integrity was confirmed by karyotyping (g-banding)(Fig. S1). hiPSCs were subsequently cultivated as described in Supplementary Material (SM).

### Differentiation of hiPSCs into 5-HT specific neurons

**Fig. 1:**
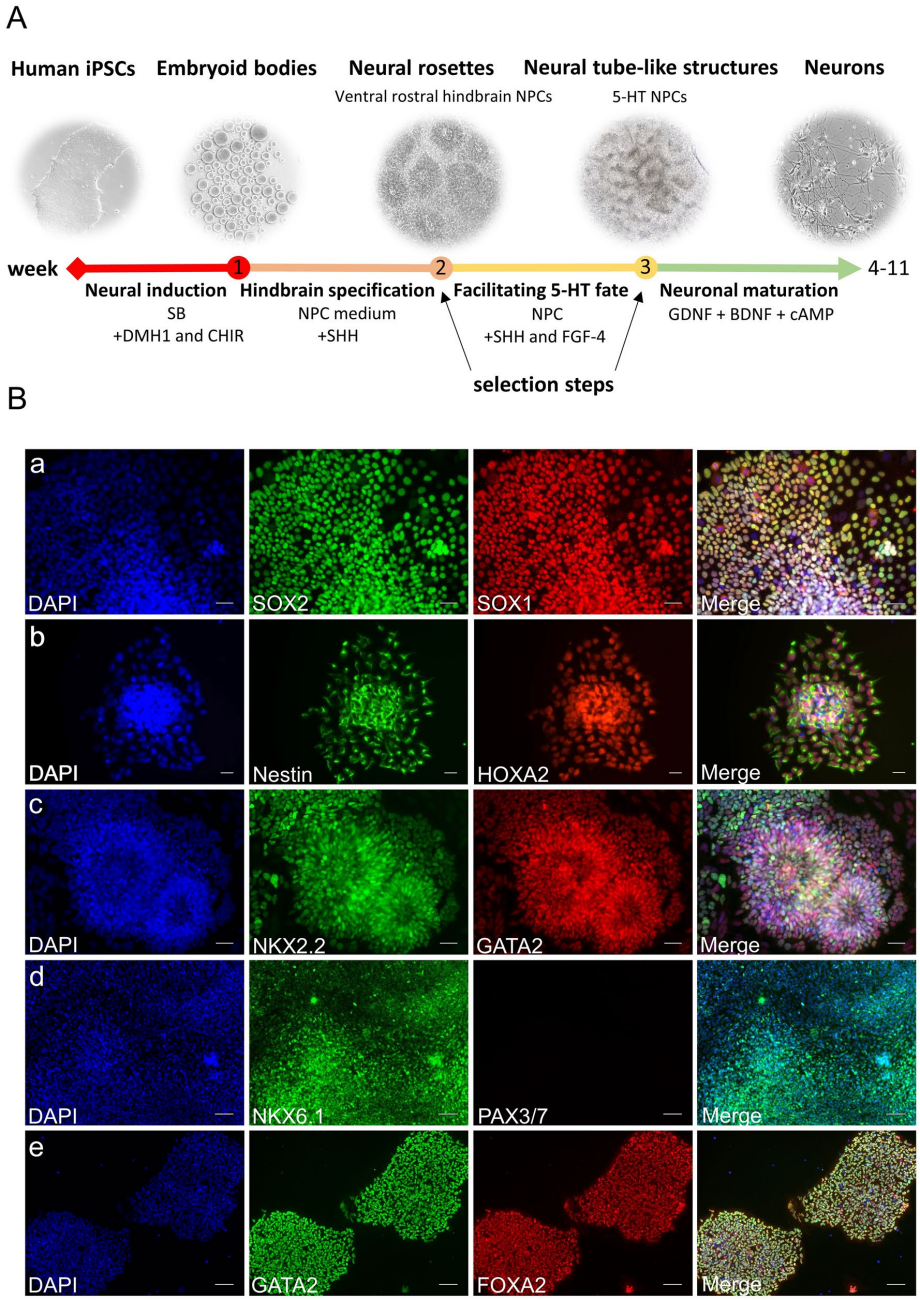
Differentiation of hiPSCs into 5-HT specific progenitors. **A** Scheme of the differentiation protocol describing the generation of hiPSCs-derived 5-HT specific neurons. **B** Generation of 5-HT specific progenitors illustrated by epifluorescence microscopy. **a**, **b** Specification of rostral hindbrain progenitors. After 1 week of neural induction cells were stained for typical neural markers, such as Nestin, SOX2, and SOX1. High expression level of HOXA2, a marker for hindbrain progenitors, was additionally found during that time point in these cells. **c**, **d** Ventralization of cells was shown by positive IF staining of NKX2.2, NKX6.1, GATA2 and negative staining of PAX3/7, when treated with 1000 ng/ml SHH for 1 week. **e** Ventralized rostral progenitors were treated with FGF4 for one further week to generate 5-HT progenitors with immunoreactivity for GATA2 and FOXA2. Scale bar: 50 μm (**a**–**c**), 100 µm (**d**, **e**). Cell nuclei were counterstained with DAPI, and Alexa Fluor 488 (SOX2, Nestin, NKX2.2, NKX6.1, GATA2), and Alexa Fluor 555 (SOX1, HOXA2, GATA2, PAX3/7, FOXA2) were used to visualize target proteins. 5-HT specific progenitor differentiation was verified using the JMUi001-A iPS line (data not shown)

**Fig. 2:**
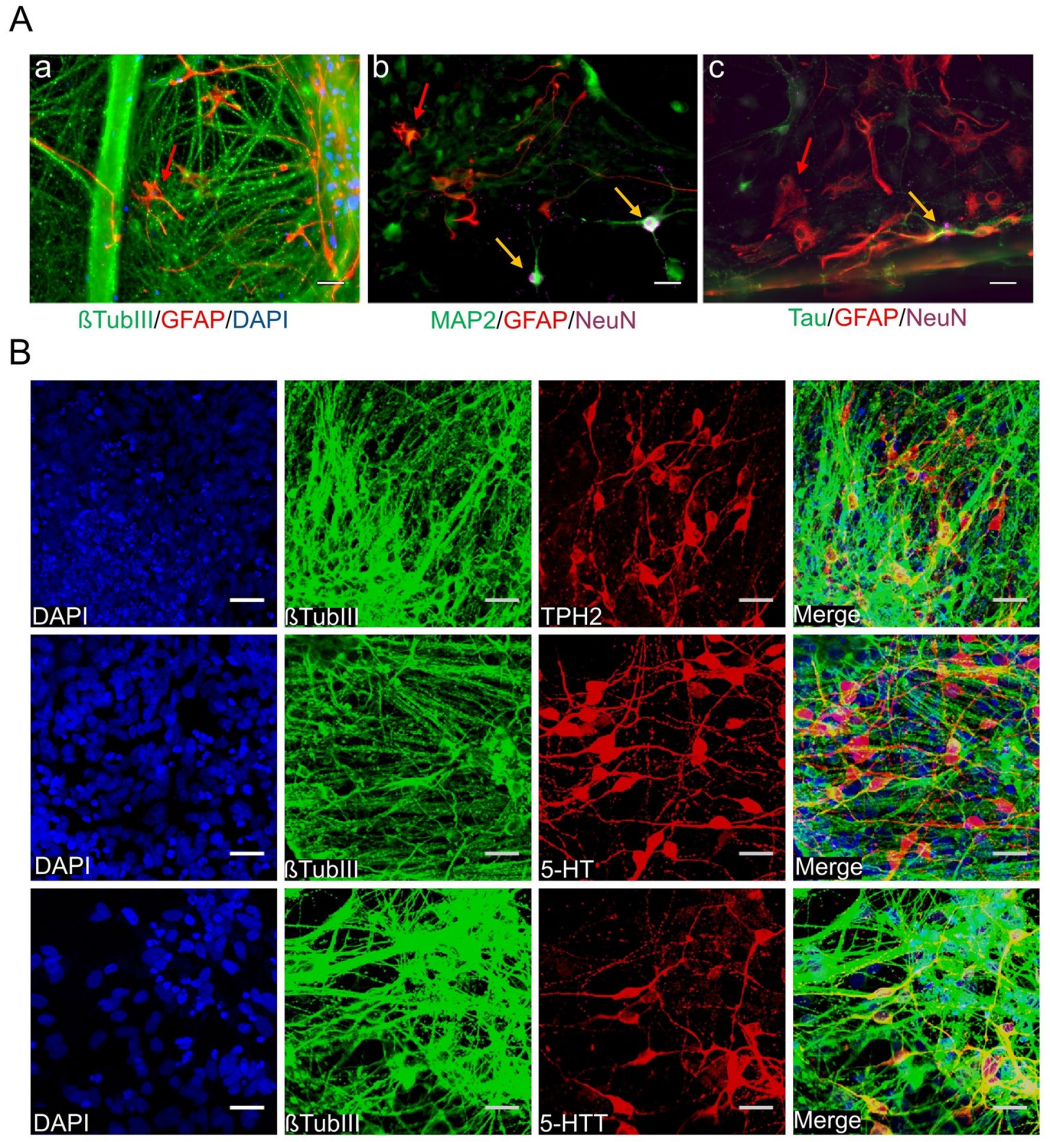
Generation of 5-HT specific neurons from human iPSC-derived 5-HT specific progenitors. **A** Our differentiation protocol generates βTUBIII + neurons (**A**, **a** stained in green) and GFAP + astrocytes (**A**, **a**–**c**, stained in red, examples marked by red arrows) following 5 weeks of neuronal maturation. 5-week-old neurons were additionally proven to be mature neurons illustrated by positive staining of (**A**, **b**) MAP2 (stained in green) and NeuN (stained in magenta) and (**A**, **c**) Tau (stained in green), and NeuN (stained in magenta). Mature neurons are marked by yellow arrows. Pictures were taken using epifluorescence microscopy. **B** All neurons were co-stained for an antibody against βTUBIII and TPH2, 5-HT, and 5-HTT after 4–5 weeks of neuronal maturation (differentiation week 7–8). Pictures were taken using confocal microscopy. Scale bar: 50 μm. Cell nuclei were counterstained with DAPI and Alexa Fluor 488 (βTUBIII, MAP2, Tau) and Alexa Fluor 555 (GFAP, TPH2, 5-HT, 5-HTT) and Alexa Fluor 647 (NeuN) were used to visualize target proteins. 5-HT specific neuronal differentiation was verified using the JMUi001-A iPS line (data not shown)

iPSC colonies were maintained on Matrigel-coated 6-well plates in StemMACS™ iPS-Brew XF (Miltenyi Biotec) and neuralized using a modified version of a previously described hindbrain differentiation protocol (Fig. 1A) (Lu et al. 2016). Briefly, hiPSCs were detached with Accutase and transferred to an ultra-low attachment plate (Corning) in neural induction medium (DMEM-F12:Neurobasal (1:1), 1 × N2 supplement (Invitrogen, Carlsbad, CA, USA), 1 × B27 supplement (Invitrogen), 1.4 µM CHIR 99021 (Axon Medchem, Groningen, The Netherlands), 2 µM DMH1 (Tocris Bioscience, Bristol, U.K.) and 2 μM SB431542 (Miltenyi Biotec)) to generate EBs. After 7 days, medium-sized (200–300 µm in diameter), free-floating EBs were transferred to poly-ornithine (PO, 20 µg/ml, Merck Millipore)- and laminin (10 µg/ml, BioLamina, Sundbyberg, Sweden)-coated 6-well plates (15 EBs/well) in neural progenitor cell (NPC) medium containing neural induction medium and SHH C25II (1000 ng/ml; R&D Systems, Minneapolis, MN, USA) for ventralization of rostral hindbrain progenitors. Rosette-forming EBs were manually isolated and plated on PO–laminin-coated 6-well plates in the same NPC medium containing SHH C25II (1000 ng/ml) and FGF4 (10 ng/ml; Peprotech, Hamburg, Germany) to specify the serotonergic fate of NPCs for one further week. The medium was changed every other day. At the beginning of week 4 of differentiation NPCs showing the right morphology (small, tightly packed cells organized in neural tube-like structures) were selected, dissociated using Accutase for 3 min and finally plated for neuronal differentiation (4–8 weeks) at lower densities (3 × 10^4^ cells/1.9 cm^2^) onto PO-laminin-coated glass coverslips for electrophysiological analysis, on poly-d-lysine (PDL)-laminin-coated 8-chamber slides (2 × 10^4^ cells/0.8 cm^2^) for immunofluorescence (IF) stainings, and on PO-laminin-coated glass slides (1 × 10^5^ cells/slide) for *d*STORM analyses in neuronal maturation medium (NMM). NMM was composed of brain-derived neurotrophic factor (BDNF, 10 ng/ml, Peprotech), glial cell-derived neurotrophic factor (10 ng/ml, Peprotech), ascorbic acid (200 nM, Sigma-Aldrich), laminin (1 μg/ml, Biolamina), 2.5 µM DAPT, 10 ng/ml insulin-like growth factor-I (IGF-I), and 1 ng/ml transforming growth factor β3 (all from PeproTech) in Neurobasal supplemented with N2 + B27. The medium was changed every third day for 4–8 weeks. Successful generation of rostral hindbrain NPCs after 1 week (Fig. 1B a–b), ventral rostral hindbrain NPCs after 2 weeks (Fig. 1B c–d), and ventral rostral hindbrain NPCs with definite serotonergic fate after 3 weeks (Fig. 1B e) was indicated by double immunostainings for respective neurodevelopmental markers. Maturation of these NPCs into 5-HT specific neurons after 5 weeks of neuronal maturation was demonstrated by IF stainings for TPH2, 5-HT, and 5-HTT (Fig. 2B).

### Imaging techniques

#### Epifluorescence

Cells were grown, fixed, and labelled on PDL-laminin-coated 8-chamber slides (20 × 10^3^ cells/well; Corning) as described in SM. Images of differentiation markers in NPCs (Fig. 1B), and images of neural maturation, and glia cell markers (Fig. 2A) were obtained using an Olympus motorized inverted system microscope IX81, an X-Cite fluorescence illuminator, and an XM10 camera (all Olympus, Leinfelden-Echterdingen, Germany). Pictures were taken at 10×, 20×, and/or 40 × magnifications through the exposure channels for Alexa Fluor 488, Alexa Fluor 555, Alexa Fluor 647, and DAPI. Images were then processed using software CellSense (Olympus), and corrected for contrast and brightness using ImageJ v2.0.0 (Schneider et al. 2012). To further elucidate the role of CDH13 for the developing human 5-HT system we performed double immunostainings for CDH13 with Nestin as general NPC marker, HOXA2 as rostral hindbrain marker, and NKX2.2 and NKX6.1 as ventral rostral hindbrain markers in 3 week old NPCs (Fig. S2A), and reassessed CDH13 immunoreactivity after 8 weeks of neuronal maturation in ßTUB + /TPH2 + cells (Fig. S2B). The proportion of CDH13 + cells among Nestin + /HOXA2 + NPCs and among ßTUBIII + /TPH2 + double-positive cells was counted in images from four randomly selected areas per coverslip (*n* = 4 coverslips per independent differentiation) using ImageJ.

#### Confocal microscopy

Cells were grown, fixed, and labelled on PDL-laminin-coated 8-chamber slides (Corning; 20 × 10^3^ cells/well, for detailed protocol, see SM). Images of neuronal subtype markers were generated using a FluoView FV1000 confocal microscope (Olympus) with a 20× UPlanSAPO, NA 0.75 (air) objective. Stack images were taken by laser illumination at 561 nm (Alexa Fluor 555), 488 nm (Alexa Fluor 488), and 405 nm (DAPI). 12-bit raw images were processed with the imaging software Fluoview, version 4.1.a (Olympus).

#### Structured illumination microscopy (SIM)

SIM microscopy was performed as previously described (Forero et al. 2017). Cells were grown, fixed, and labelled on PO-laminin-coated 24-well plates (3 × 10^4^ cells/well) as described in SM. Images of CDH13 expression in TPH2 + neurons were captured with a commercial inverted SIM microscope (Zeiss ELYRA, Carl Zeiss Microscopy, Oberkochen, Germany) using an oil-immersion objective (Plan-Apochromat 6x/1.4 Oil Dic M27) (Gustafsson 2000; Wegel et al. 2016). Excitation of the fluorophores was performed by laser illumination at 642 nm (Alexa Fluor 647), 561 nm (Alexa Fluor 555), 488 nm (Alexa Fluor 488), and 405 nm (DAPI), and fluorescence light was filtered by appropriate detection filters: LP 655 (Alexa Fluor 647), BP 570–620 + LP 750 (Alexa Fluor 555), BP 495–550 + LP 750 (Alexa Fluor 488), and BP 420–480 + LP 750 (DAPI).

#### *Direct* stochastic optical reconstruction microscopy (*d*STORM)

For *d*STORM, hiPSC-derived neurons were grown, fixed, and labelled on PO-laminin-coated glass bottom dishes (ibidi, Munich, Germany) as described in SM. For fluorophore photo switching, a buffer containing 1% glucose and 100 mM β-mercaptoethylamine (Sigma-Aldrich) in PBS adjusted to a pH of 8.0 was used. *d*STORM was performed on a wide-field setup for localization microscopy (van de Linde et al. 2011). A diode laser with a wavelength of 640 nm (iBeam smart, TOPTICA Photonics, Munich, Germany; maximum power of 200 mW) and a diode-pumped solid-state laser with a wavelength of 532 nm (gem, Laser Quantum, Stockport, U.K. maximum power of 500 mW) were used for excitation of Alexa Fluor 647 and Alexa Fluor 532 respectively. Laser beams were cleaned-up by bandpass filters (Semrock, NY, USA) and combined by appropriate dichroic mirrors (LaserMUX filters, Semrock). Afterwards they were focused onto the back focal plane of the high numerical oil-immersion objective (alpha Plan-Apochromat 100x/1.46 Oil DIC), which is part of an inverted fluorescence microscope (Zeiss Axio Observer.Z1, Carl Zeiss Microscopy) equipped with an autofocus system (Definite Focus, Carl Zeiss Microscopy). To separate the excitation light from the fluorescence light, a suitable dichroic beam splitter (Semrock) was placed into the light path before the laser beams enter the objective. Fluorescence light of Alexa Fluor 647 and Alexa Fluor 532 was collected by the objective and splitted by a dichroic mirror (Chroma, Bellows Falls, Vermont, USA) to two separate EMCCD cameras (iXon Ultra 897, Andor Technology, Belfast, U.K.). Before entering the cameras, it was filtered by appropriate detection filters (Semrock/Chroma). For every image, 15 × 10^3^ frames were taken with an integration time of 10 ms per frame. Data analysis was performed using ThunderSTORM (Ovesny et al. 2014). Gold beads were used for drift correction.

### Electrophysiological recordings

Single-cell patch-clamp recordings were performed to investigate functional maturation of hiPSC-derived 5-HT specific neurons. Cultured neurons were collected from 24-well culture plates and whole-cell recordings of at least 10 neurons per independent experiment (*n* = 3) were performed weekly over a period of 6 weeks at room temperature in a bath solution consisting of 135 mM NaCl, 5.4 mM KCl, 1.8 mM CaCl_2_, 1 mM MgCl_2_, 10 mM glucose, 5 mM HEPES, pH 7.4 as previously reported (Hamill et al. 1981). Patch pipettes were pulled from borosilicate glass capillaries (DWK Life Sciences, Wertheim, Germany) and heat-polished to give input resistances of 2–5 MΩ (whole-cell). The pipette recording solution contained 120 mM potassium methansulfonate (CH_3_KO_3_S), 4 mM NaCl, 1 mM MgCl_2_, 0.5 mM CaCl_2_, 10 mM ethylene-bis(oxyethylenenitrilo) tetraacetate (EGTA), 3 mM ATP-Mg, 0.3 mM GTP-TRIS, and 10 mM HEPES (pH 7.2). Currents were recorded with an EPC9 (HEKA, Lambrecht/Pfalz, Germany) patch-clamp amplifier and low pass-filtered at 2 kHz. Stimulation and data acquisition were controlled by the PULSE/PULSEFIT software package (HEKA) on a Macintosh computer, and data analysis was performed with IGOR software (WaveMetrics, Lake Oswego, OR, USA). Properties of 5-HT specific neurons were analyzed according to existing electrophysiological criteria (de Kock et al. 2006; Li et al. 2001; Vandermaelen and Aghajanian 1983), such as amplitude, half-height width (HHW) and action potential frequency. Our main criteria for 5-HT specific neuron identity were action potential HHW of > 1.5 ms and maximal sustained firing rate of < 12 Hz (Mlinar et al. 2015).

### Statistical analyses

Except of qPCR analysis (*n* = 2), all experiments described in this study have been conducted in a total of three independent differentiations (*n* = 3). Values were expressed as mean ± s.e.m. Differences between means were assessed by one-way analysis of variance (ANOVA) and *P* < 0.05 was considered as significant.

## Results

### Hindbrain progenitors differentiate into a mixed culture of specific neuronal subtypes

**Fig. 3:**
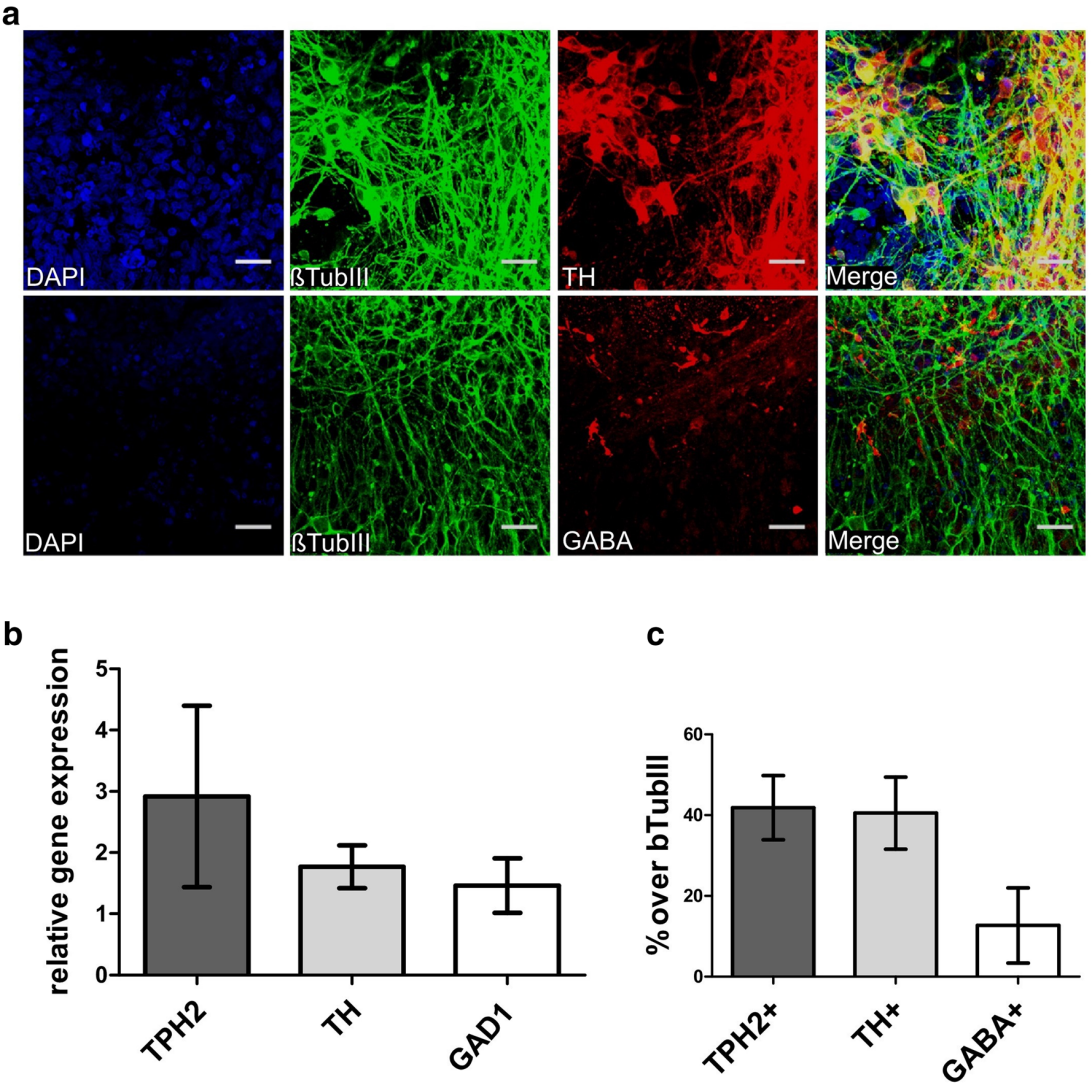
Specification of different neuronal subtypes. **a** Neurons were co-stained for an antibody against βTUBIII, TH, and GABA after 4–5 weeks of neuronal maturation (differentiation week 7–8). Pictures were taken using confocal microscopy. Scale bar: 50 μm. Cell nuclei were counterstained with DAPI and Alexa Fluor 488 (βTUBIII) and Alexa Fluor 555 (TH, GABA) were used to visualize target proteins. **b** Relative gene expression levels of the human iPSC-derived 5-HT specific (TPH2 +), catecholaminergic (TH +) and GABAergic (GAD1 +) neurons (*n* = 2 independent differentiations). **c** Quantification of the amount of 5-HT specific (TPH2 +), catecholaminergic (TH +) and GABAergic (GABA +) neurons within the neuronal cell culture system (*n* = 3 independent differentiations)

After 5 weeks of neuronal maturation, most of the cells developed into βTUBIII + neurons (> 70%) and only a very low percentage of glial fibrillary acidic protein positive (GFAP +) astrocytes (Fig. 2A, a). Additionally, these 5-week-old neurons were proven to already be mature by positive staining of dendritic marker microtubule-associated protein 2 (MAP2), axonal marker Tau protein and neuronal nuclear protein (NeuN) (Fig. 2a, b, c). Among the βTUBIII + cells around 42% of the neurons in our culture system displayed a serotonergic phenotype illustrated by a positive TPH2 (TPH2 +) staining. To determine specific neuronal subtypes in our neuronal cell culture system we performed IF of neuronal subtype markers (Fig. 3a) and counting analyses (Fig. 3c). These investigations revealed that in addition to the 42% of 5-HT specific neurons, 40% of all the neurons showed a catecholaminergic and 12% a GABAergic phenotype. mRNA expression of subtype markers *TPH2*, *TH,* and *GAD1* were shown by qRT-PCR (Fig. 3b).

### hiPSC-derived 5-HT specific neurons display phenotypes of both median and dorsal raphe 5-HT specific neurons

**Fig. 4:**
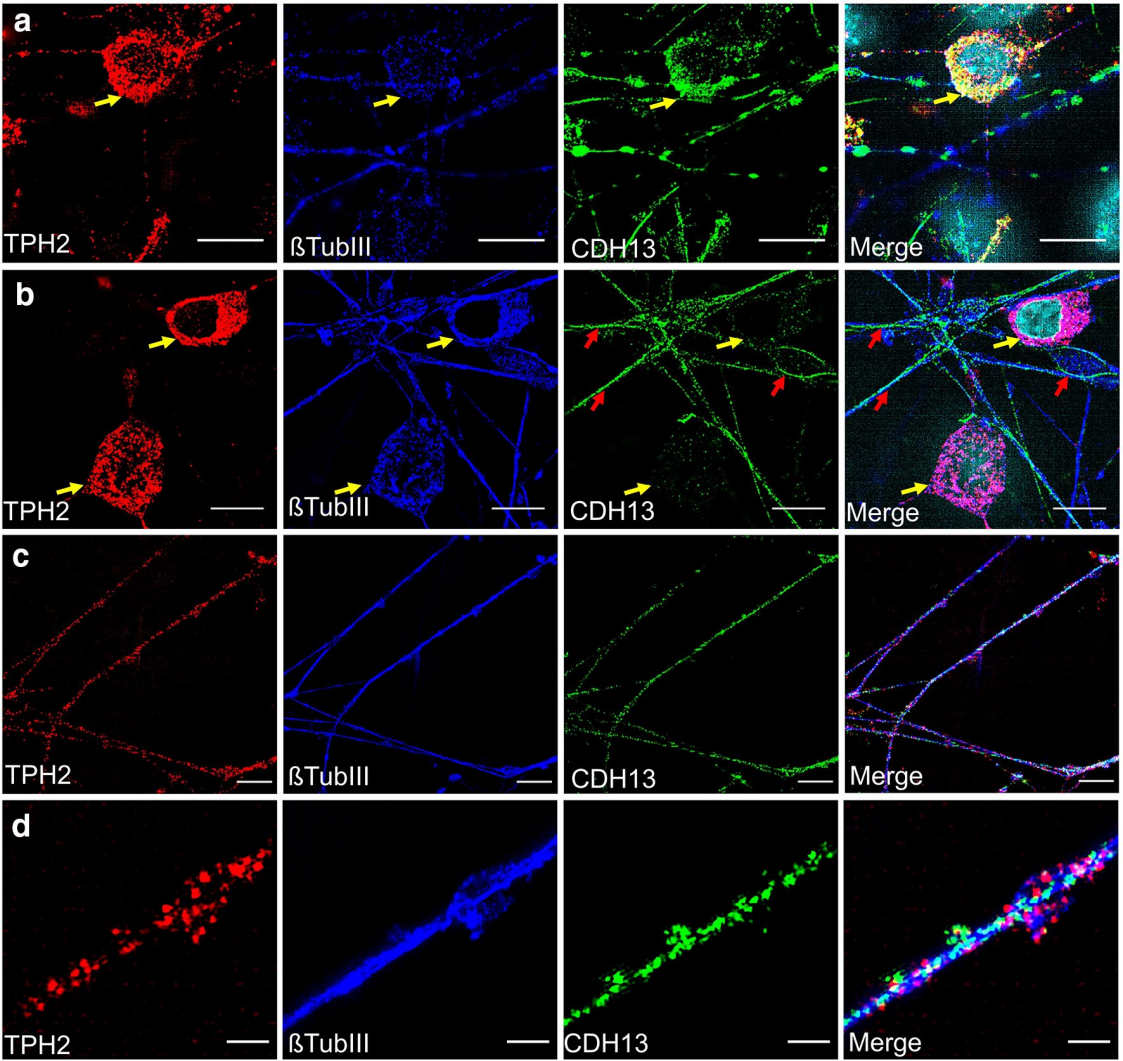
CDH13 expression in TPH2 + neurons using SIM. **a** IF staining of a TPH2 + neuron which is positive for CDH13 (yellow arrows). **b** IF staining of TPH2 + neurons which are negative for CDH13 (yellow arrows). Additionally, βTUBIII + fibers that are negative for TPH2 are immunoreactive for CDH13 and marked by a red arrow. **c** IF staining of TPH2 + fibers being positive for CDH13. **d** Close-up image of (**c**). Scale bar: **a**, **b**, **c** 10 μm; **d** 2 µm. Alexa Fluor 488 (βTUBIII), Alexa Fluor 555 (TPH2) and Alexa Fluor 647 (CDH13) were used to visualize target proteins

Due to the importance of CDH13 for neurodevelopmental disorders, the expression of this protein was analyzed in hiPSC-derived 5-HT specific neurons using epifluorescence microscopy and SIM. Our findings indicated that CDH13 was expressed in a subset of the TPH2 + neurons (Figs. 4, S2B) but was lacking from the majority of 5-HT specific neurons identified in our culture (Fig. 4b). Among Nestin + /HOXA2 + NPCs the proportion of CDH13 + cells was only 1.5%. These CDH13 + NPCs build small cell clusters which occur only sparsely (Fig. S2A). However, after 8 weeks of neuronal maturation CDH13 immunoreactivity was evident equally across the whole culture with about 40% of ßTUBIII + /CDH13 + cells being also CDH13-positive (Fig. S2B). We observed the same expression pattern previously detected in murine 5-HT specific neurons, with CDH13 outlining both the soma and extending neurites (Fig. 4a, c, d). A three-dimensional reconstruction of these CDH13 + 5-HT specific neurons suggested a potential role in cell–cell interaction, showing two TPH2 + neurons in close proximity with CDH13 expression at the contact region between them (Supplementary video 1).

### Electrophysiological signature of hiPSC-derived 5-HT specific neurons

**Fig. 5:**
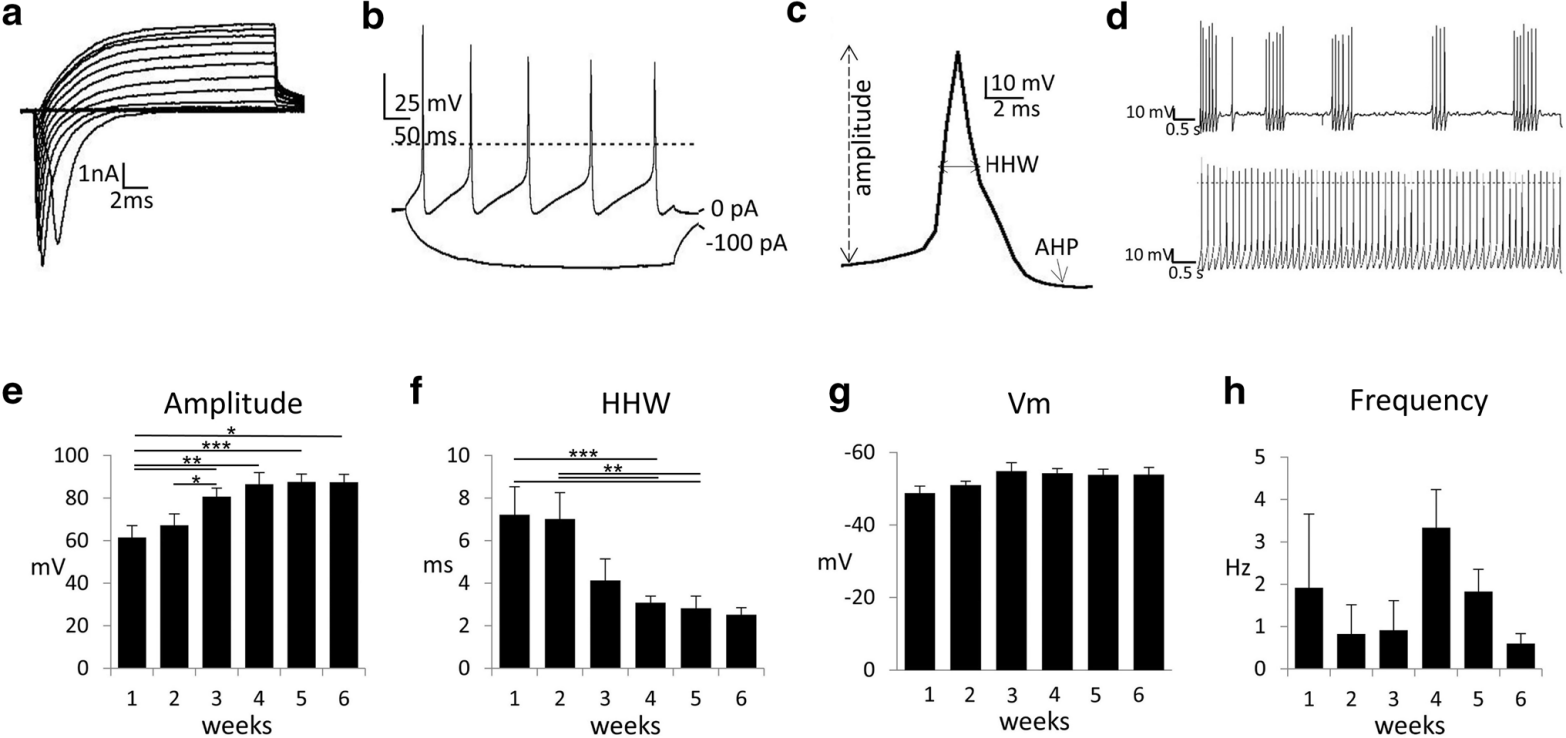
Electrophysiological properties of human iPSC-derived 5-HT specific neurons. **a** Voltage-gated Na + and K + currents triggered by voltage steps from -100 to + 70 mV at a holding potential of − 80 mV. **b** Action potentials were induced upon injection of current steps from − 120 to + 120 pA. Depicted are traces at -100 pA and at 0 pA with spontaneous action potentials. **c** Single action potential on an expanded time scale. Indicated are amplitude, HHW and afterhyperpolarization (AHP). **d** Neurons display two typical modes of activity: bursts with intercalated pauses (upper trace) or activity with constant frequency (lower trace). **e**–**h** Bar graphs of electrophysiological properties of human iPSC-derived 5-HT specific neurons over a time period of 6 weeks. **e** AP amplitude, **f** half-height width (HHW), **g** resting membrane potential and **h** AP frequency. Electrophysiological signature characteristic of serotonergic neuron was verified using the JMUi001-A iPS line (data not shown). Pairwise post-hoc t-tests, * *P* < .05, ** *P* < .01, *** *P* < .001

Voltage steps from − 100 to + 70 mV generated inward Na^+^ and outward K^+^ currents (Fig. 5a), responsible for the induction of action potentials. Current injections from − 120 to + 120 pA elicited action potentials (Fig. 5b). Generated neurons displayed typical 5-HT specific neuron identity proven by action potential HHW of > 1.5 ms (Fig. 5c) and maximal sustained firing rate of < 12 Hz (Fig. 5d, h). During the first 4 weeks of maturation the amplitude of the action potentials increased significantly (Fig. 5e: amplitude: w1: 61.52 ± 5.57 mV, *n* = 14; w2: 67.18 ± 5.42 mV, *n* = 16; w3: 80.67 ± 3.98 mV, *n* = 22; w4: 86,56 ± 5.41 mV, *n* = 22; w5: 87.62 ± 3.64 mV, *n* = 17; w6: 87.48 ± 3.71 mV, *n* = 5; F(5,90) = 4.538, *P* = 0.001), whereas the duration of action potentials decreased (Fig. 5f: HHW: w1: 7.22 ± 1.31 ms, *n* = 14; w2: 7.02 ± 1.24 ms, *n* = 16; w3: 4.13 ± 1.01 ms, *n* = 22; w4: 3.09 ± 0.3 ms, *n* = 22; w5: 2.83 ± 0.57 ms, *n* = 17; w6: 2.52 ± 0.34 ms, *n* = 5; F(5,90) = 4.453, *P* = 0.0011). The membrane potential of the neurons did not change significantly over time (Fig. 5g: Vm: w1: − 48.75 ± 1.98 mV, *n* = 14; w2: − 50 ± 1.14 mV, *n* = 16; w3: − 54.85 ± 2.37 mV, *n* = 22; w4: − 54.29 ± 1.28 mV, *n* = 22; w5: − 53.88 ± 1.52 mV, *n* = 17; w6: − 53.9 ± 1.94 mV, *n* = 5; F(5,90) = 1.592, *P* = 0.1705) as well as the spike frequency (Fig. 5h: frequency: w1: 1.92 ± 1.74 Hz, *n* = 4; w2: 0.83 ± 0.69 Hz, *n* = 5; w3: 0.92 ± 0.7 Hz, *n* = 8; w4: 3.34 ± 0.9 Hz, *n* = 15; w5: 1.83 ± 0.52 Hz, *n* = 12; w6: 0.6 ± 0.23 Hz, *n* = 3; F(5,41) = 1.447, *P* = 0.2283).

### Super-resolution imaging of synaptic connectivity

**Fig. 6:**
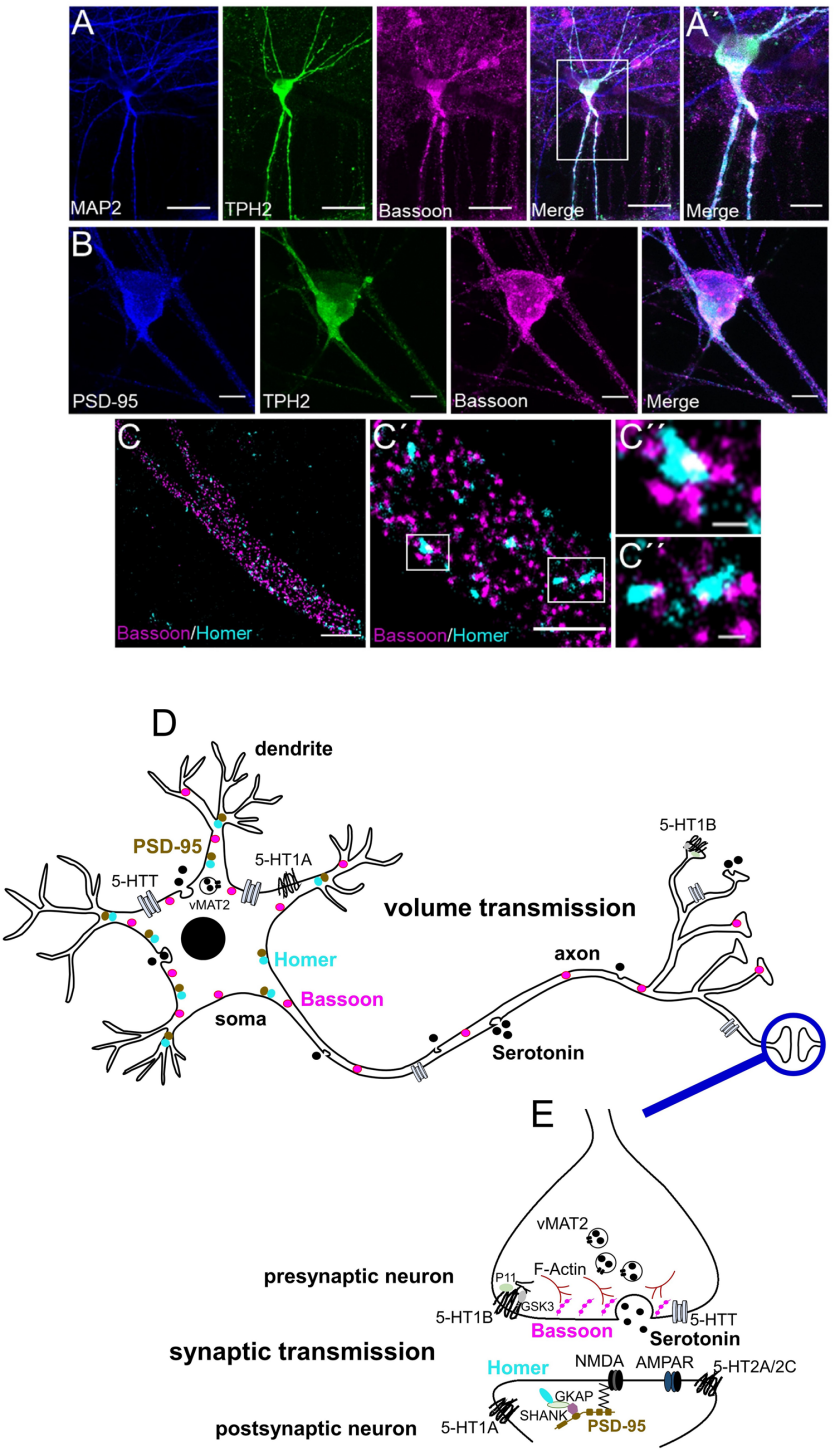
Human iPSC-derived 5-HT specific neurons function rather via somatodendritic 5-HT release than synaptic transmission. **A** The presynaptic marker Bassoon is evenly expressed along soma and extensions of TPH2 + human iPSC-derived neurons. **A**´ Close-up image of (**A**). **B** Bassoon is localized in close proximity to the postsynaptic marker PSD-95 in dendrites and cell bodies of TPH2 + neurons. **C** *d*STORM revealed a higher expression of Bassoon compared to the postsynaptic marker Homer in TPH2 + neurons. **C**´ Close-up images of (**C**). **C**´´ Example images illustrating contact points between Homer and Bassoon. Simplified schematic illustrations of Bassoon, PSD-95 and Homer expression in (**D**) volume transmission and (**E**) synaptic transmission. Scale bar: **A** 30 µm; **A**´ 10 µm; **B** 10 µm; **C** 2 μm; **C**´ 1 µm and **C**´´ 150 nm. Alexa Fluor 488 (PSD-95; MAP2), Alexa Fluor 532 (Homer; TPH2) and Alexa Fluor 647 (Bassoon) were used to visualize target proteins

To further evaluate synapse formation and connectivity among the differentiated 5-HT specific neurons, we performed confocal microscopy and *d*STORM, a super-resolution microscopy technique to visualize the presence of synaptic structures (Fig. 6). Here, neurons immunoreactive for TPH2 were additionally analyzed for the expression of synaptic proteins. Pre-and postsynaptic structure and machinery was confirmed by imaging Bassoon, a marker for the presynaptic active zone, together with the major scaffolding protein in the excitatory postsynaptic density (PSD) PSD-95 or Homer, a PSD scaffolding protein. As shown in Fig. 6A, B, Bassoon was not only expressed in axon terminals, but was also evenly distributed along the soma and extension of TPH2 + 5-HT specific neurons. Interestingly, these neurons showed Bassoon expression in Map2 + dendrites (Fig. 6A) and in close proximity to PSD-95 (Fig. 6B). We implemented Alexa Fluor 647 for Bassoon and Alexa Fluor 532 for the labeling of Homer as a suitable combination for the two-color *d*STORM analysis (Fig. 6C). TPH2 + neurons were targeted for presynaptic Bassoon, as well as postsynaptic Homer, which led to the identification of an overall more pronounced expression of Bassoon compared to Homer (Fig. 6C to C´´). However, even when close proximity of contact points between Homer and Bassoon was observed (Fig. 6C´´), no typical bar structures illustrating synaptic transmission were identified. Generated 5-HT specific neurons thus appear more likely to function via somatodendritic 5-HT release (Fig. 6D) rather than via synaptic transmission (Fig. 6E).

## Discussion

This study reports the generation 5-HT specific neurons from hiPSCs. In addition to a high percentage of 5-HT specific neurons (~ 42%), also catecholaminergic (~ 40%) and GABAergic neurons (~ 12%) were generated. Immunohistochemistry confirmed neuronal subtype-specific marker expression, while high-end fluorescence microscopy and whole-cell patch clamp recordings illustrated the synaptic compartment of human 5-HT specific neurons and revealed characteristic electrophysiological features.

In our first attempts to generate functionally active 5-HT specific neurons from hiPSCs we strictly followed the protocol published by Lu and associates which is based on an adherent culture system (Lu et al. 2016). An efficiency of more than 60% 5-HT specific neurons was reported for this approach. However, during our undertaking to replicate the differentiation efficiencies the adherent system resulted in a low percentage of neuronal cells (5–10%). Subsequently, we adjusted the protocol to an EB formation system (Fig. 1A) as we attributed the loss of neural progenitors to repetitive splitting during the neural progenitor expansion steps. The applied small molecules and time course of their administration were maintained as in the original protocol. In our alternative EB formation approach, we opted for positive selection of cells by carefully lifting neural rosettes after 1 week of ventralization by SHH and selecting neural tube-like structures following one further week of 5-HT fate specification (SHH + FGF4). Thereby, we avoided splitting cells during the entire differentiation process, which resulted in a nearly pure neuronal culture. Finally, these adjustments enriched the cell population to 42% 5-HT specific neurons within the overall number of neuronal cells. Recently, a similar adaptation of the original protocol (Lu et al. 2016) likewise with implementation of an EB formation system with efficiencies of more than 70% was reported (Farrelly et al. 2019; Vadodaria et al. 2019). Thus, it can be argued that initiation of serotonergic differentiation in suspension culture is a reasonable approach if adherent methods do not result in good differentiation efficiencies. This is in line with the finding that neural induction by dual SMAD inhibition (also used by our protocol with small molecules DMH1 and SB) in free-floating EBs results in an even higher proliferative capacity and differentiation potential with resulting NPCs expressing higher amounts of SHH and Nestin, when compared to an adherent culture system (Pauly et al. 2018). However, the inconsistencies of an EB system with variation in size of generated aggregates may have been one reason why we continued to gain less 5-HT specific neurons compared to the previously published protocols (Farrelly et al. 2019; Lu et al. 2016; Vadodaria et al. 2019). Since it is challenging to control the development of cells within these aggregates, EBs are known to display a complex differentiation system. A wide range of EB sizes and geometries is likely as EB cultures favor further aggregation (Dang et al. 2002). Therefore, we only picked medium-sized round EBs (200–300 µm in diameter) and plated them on PO/laminin-coated plates to allow rosette formation. Another reason why we gained a lower proportion of 5-HT specific cells than similar EB-based protocols (Farrelly et al. 2019; Vadodaria et al. 2019) might be that we seeded NPCs directly after the 3-week differentiation period, whereas the former suggest propagating NPCs for some passages at high density under adherent conditions which might stabilize the cells’ serotonergic profile.

The apparent shortcoming of a comparatively (Farrelly et al. 2019; Lu et al. 2016; Vadodaria et al. 2019) less pure serotonergic culture with our protocol might be outweighed by the advantage of a mixed neuronal culture system facilitating the investigation of interactions between different neuronal subtypes. Overall, we achieved a balanced neuronal culture of 5-HT specific (~ 42%) and catecholaminergic (~ 40%) neurons. Of note, the interaction of 5-HT specific neurons with catecholaminergic (dopaminergic and noradrenergic) neurons is of relevance for the pathogenesis of neurodevelopmental conditions (Garcia et al. 2019; Kapur and Remington 1996; Oades 2008) and has been examined in several animal-based studies (Daw et al. 2002; Wong et al. 1995). Thus, our differentiation protocol creates a model system that may be practical to further study the interaction of human 5-HT specific and catecholaminergic neurons (for review, Di Giovanni et al. 2010; Monti and Jantos 2008; Niederkofler et al. 2015). The identification of different neuronal subpopulations is in line with previous findings of catecholaminergic and GABAergic neurons among 5-HT specific neurons obtained by the differentiation method under investigation (Lu et al. 2016).

The original protocol generated a culture of high-density median raphe 5-HT specific neurons (Lu et al. 2016). Converging evidence, however, suggests differences between median and dorsal raphe 5-HT specific neurons with respect to their electrophysiological properties (Beck et al. 2004) and functional connectivity (Beliveau et al. 2015). Moreover, dorsal and median raphe nuclei have been ascribed differential roles for pro-social, aggressive (Balazsfi et al. 2018), and addictive behavior (Verheij et al. 2018). Therefore, we examined our approach for subdivision into median and dorsal raphe 5-HT specific neurons. We recently demonstrated that CDH13 contributes to the migration of 5-HT specific neurons to distinct subregions of the raphe nuclei, preferentially the lateral wing of the dorsal raphe (Andrea Forero et al. 2017) and has been associated with various neurodevelopmental disorders including ADHD (Lasky-Su et al. 2008; Lesch et al. 2008; Neale et al. 2010) and ASD (Sanders et al. 2011, 2015). SIM microscopy of our 5-HT specific neurons indicates that CDH13 is highly expressed in a subset of cultured TPH2 + neurons. Whereas only 1.5% of 5-HT specific NPCs expressed CDH13, neuronal maturation led to an increase in CDH13 + 5-HT specific cells with about 40% of TPH2 + /ßTUBIII + neurons displaying immunoreactivity for CDH13 + after 8 weeks of neuronal maturation. This time-dependent increase in CDH13 + cells between 5-HT specific neurogenesis and maturation further strengthens the view that CDH13 is involved in the development of the 5-HT system (Forero et al. 2020). We conclude that differentiation may generate a mixture of median and dorsal raphe 5-HT specific neurons, and we provide evidence that proteins which have been identified to influence the risk for neurodevelopmental disorders may be studied using this cell system. However, further investigation including additional markers for these various subsets of raphe nuclei in human-derived cells is necessary to eventually distinguish cells with rhombomere 1 vs. rhombomere 2/3 specification which give rise to dorsal and median raphe neurons, respectively (Alonso et al. 2013; Bang et al. 2012; Jensen et al. 2008).

To analyze neuronal maturity we stained for markers of neuronal maturation, such as the dendritic marker MAP2, the axonal marker Tau, and NeuN, a marker labeling maturing neurons. Interestingly, already after 5 weeks of neuronal maturation generated neurons were expressing these markers. Thus, our differentiation protocol enables a rapid and efficient generation of fully mature neurons and also yields a distinct proportion of astrocytes that are known to support neuron function. Therefore, the astrocytes possibly benefit those generated neurons in vitro and accelerate maturation. However, to further prove reliable differentiation of hiPSCs into 5-HT specific neurons that form synaptic connections and show the characteristic features of functional in vivo 5-HT specific neurons, we examined our cells using electrophysiological techniques as well as *d*STORM.

To assess functional maturation of 5-HT specific neurons, whole-cell recordings were performed weekly over a period of 6 weeks. These measurements revealed typical ion currents, repetitive firing elicited by current injections and spontaneous firing in neurons detectable already after 1 week of neuronal maturation. Interestingly, the rate of maturation and firing properties were similar within experiments and over time. Using electrophysiological properties for identifying neurons as 5-HT specific was previously discussed (Vandermaelen and Aghajanian 1983). Some of the applied criteria, such as input resistance or afterhyperpolarisation lead to high error rates in identifying 5-HT specific neurons as was demonstrated by Calizo and colleagues (Calizo et al. 2011). The lowest error rates were shown for action potential duration. In this study, the frequency of measured action potentials varied over time, but always matched the criteria for 5-HT neuron specification and was less than 12 Hz. Over the whole time period measured spontaneous action potential firing was evident. During the 6-week course, action potentials rose and got faster as the amplitude increased and the HHW decreased. After 4 weeks of maturation no further changes of the neurons’ electrophysiological properties occurred. The frequency of action potentials remained between 0.5 and 4 Hz, consistently meeting criteria of 5-HT specific neurons, in line with the findings of Lu and associates (Lu et al. 2016) who described an average firing frequency of 2.9 Hz. Our longitudinal electrophysiological data indicate that functional maturation of hiPSC-derived 5-HT specific neurons requires at least 4 weeks of outgrowth in NMM. Of note, we found two modes of firing activity: regular clock-like tonic activity with constant frequency and burst-firing activity. It is discussed that the regular firing pattern is associated with a constant basal tonic 5-HT release by somatodendritic volume transmission, whereas burst firing leads to phasic synaptic neurotransmission (Gartside et al. 2000; Quentin et al. 2018). Hence, our electrophysiological findings are a first indicator that these two modes of neurotransmission might also occur in hiPSC-derived 5-HT specific neurons. HHW and frequency of action potentials are sensitive indicators for 5-HT specific neurons, but cell types might not be distinguished by their electrophysiological signatures alone.

Therefore, we additionally confirmed synaptic connectivity among differentiated 5-HT specific neurons using confocal microscopy and the super-resolution microscopy technique *d*STORM which allows to precisely visualize the existence of synaptic structures (Heilemann et al. 2008, van de Linde et al. 2011). 8-week-old TPH2 + neurons express Bassoon, a marker for the presynaptic active zone, as well as PSD-95 and Homer, the postsynaptic density scaffolding protein (Bresler et al. 2004; Dresbach et al. 2006; Tao-Cheng et al. 2014). Bassoon as a marker for the active zone was, therefore, used to identify where hiPSC-derived 5-HT specific neurons form synapses with the property to organize neurotransmitter release. However, we observed that Bassoon is not exclusively restricted to axon terminals as seen in hippocampal neurons, but is evenly distributed throughout the cell, and that there is a lack of coinciding pre-and postsynaptic bar structures. This supports the assumption that our in vitro 5-HT specific neurons communicate through non-synaptic axonal and somatodendritic release, in addition to synaptic transmission (Adell et al. 2002; De-Miguel and Trueta 2005; Lau et al. 2010; Vizi et al. 2004).

The results of our study indicate that an EB formation system can be alternatively used if an adherent protocol does not result in reliable ventral hindbrain induction for the generation of 5-HT specific neurons from hiPSCs. As a limitation, it has to be noted that our study included only two hiPSC lines. Therefore, an EB-based serotonergic differentiation method might not be a better option than the adherent protocol in all cell lines. Hence, we suggest researchers to test both approaches to find the individually best fitting solution which might not only rely on characteristics of different cell lines but also on inter-lab variability and personal handling. Our approach generates mature and electrophysiologically active median and dorsal 5-HT specific neurons, side-by-side with catecholaminergic and a smaller portion of GABAergic neurons. The distribution of synaptic proteins alongside the neurons’ soma and their axonal and neuritic extensions corroborates the importance of extrasynaptic “volume” transmission in the human 5-HT system. Additionally, our data substantiate the relevance of CDH13 for the 5-HT system. The described cell culture model is, therefore, a practical tool to specifically study the development of human 5-HT specific neurons and their interplay with other neuronal subtypes. Especially the investigation of NPC subtype markers alongside identified but not yet functionally studied potential risk genes emerging from GWAS findings, is a promising approach for further expediting the molecular underpinnings of neuropsychiatric disorders. The replication and refinement of protocols for the differentiation of neurons from hiPSCs, however, will remain a critical and fundamental challenge for the valid production of patient-derived cellular models of neurodevelopmental diseases and underlying genetic mechanisms.

## Supplementary Information

Below is the link to the electronic supplementary material.

Supplementary file1 Supplementary Material includes detailed methods, two tables, three figures, and one video, and can be found with this article online (DOCX 32 KB)

Supplementary file2 Supplementary Video 1. 3D reconstruction of double IF of TPH2 and CDH13. Movies illustrating 3D reconstruction using Imaris. TPH2+ neurons are labeled in green (Alexa Fluor 555) and CDH13 immunofluorescence is represented in red (Alexa Fluor 647) (MP4 11319 KB)

Supplementary file3 Supplementary Fig. S1. Characterization of hiPSC lines. hiPSC lines (A: UKWMPi001-B, B: JMUi001-A) used in this study were gained by reprogramming of fibroblasts (a), showed the typical ES-like morphology in adherent culture (b), and built EBs in suspension culture (c) (only shown for line UKWMPi001-B). Both cell lines expressed pluripotency markers as assessed by immunostaining (d) and quantitative measurements as demostrated by qRT-PCR (A, d`) or FACS analysis (B, d`). EBs spontaneously differentiated into cells of the 3 germ layers (e). Chromosomal integrity was proven by karyotyping (f). Further description of the cell lines is given in the resource tables (JPG 3246 KB)

Supplementary file5 Supplementary Fig. S2. CDH13 expression in 5-HT specific NPCs (A) and neurons (B). Representative epifluorescence imaging of CDH13 expression in NPCs (A) shows that CDH13 is expressed in only few 5-HT specific NPCs. The serotonergic fate of these NPCs is depicted by expression of the general NPC marker Nestin together with the rostral hindbrain marker HOXA2 as well as the ventral rostral hindbrain markers NKX2.2 and NKX6.1. After 8 weeks of neuronal maturation CDH13 immunoreactivity is spread equally across the whole culture with about 40% of TPH2+ and ßTUB3+ cells showing immunoreactivity for CDH13 as depicted in representative images (B). Scale bar: 50 μm (JPG 4056 KB)
