# Supplementary material for: Serotonin-specific neurons differentiated from human iPSCs form distinct subtypes with synaptic protein assembly
Source: J Neural Transm (Vienna). 2021 Feb 9;128(2):225–41. doi: 10.1007/s00702-021-02303-5 (PMC7914246; doi:10.1007/s00702-021-02303-5)
Supplement: Supplementary file 1 — Supplementary file1 Supplementary Material includes detailed methods, two tables, three figures, and one video, and can be found with this article online (DOCX 32 KB) [file 702_2021_2303_MOESM1_ESM.docx]

**Serotonin-specific neurons differentiated from human iPSCs form distinct subtypes with synaptic protein assembly**

**- Supplementary Material -**

Charline Jansch ^1,*^, Georg C. Ziegler ^1, 2,*^ Andrea Forero ^1^, Sina Gredy ^3, 4^, Sina Wäldchen ^5^, Maria Rosaria Vitale ^1, 4^, Evgeniy Svirin ^1, 4^, Johanna Zöller ^1, 6^, Jonas Waider ^1^, Katharina Günther ^7, 8^, Frank Edenhofer ^7^, Markus Sauer ^5^, Erhard Wischmeyer ^1, 3^, Klaus-Peter Lesch ^1, 4, 6^

^1^ Division of Molecular Psychiatry, Center of Mental Health, University of Würzburg, Würzburg, Germany.

^2^ Department of Psychiatry, Psychosomatics and Psychotherapy, Center of Mental Health, University of Würzburg, Würzburg, Germany.

^3^ Institute of Physiology, Molecular Electrophysiology, University of Würzburg, Würzburg, Germany

^4^ Laboratory of Psychiatric Neurobiology, Institute of Molecular Medicine, Sechenov First Moscow State Medical University, Moscow, Russia.

^5^ Department of Biotechnology and Biophysics, Biocenter, University of Würzburg, Würzburg, Germany.

^6^ Department of Translational Neuroscience, School for Mental Health and Neuroscience (MHeNS), Maastricht University, Maastricht, The Netherlands.

^7^ Department of Genomics, Stem Cell Biology and Regenerative Medicine, Institute of Molecular Biology & CMBI, Leopold-Franzens-University Innsbruck, Innsbruck, Austria.

^8^ Institute of Molecular Regenerative Medicine, SCI-TReCS, Paracelsus Medical University, Salzburg, Austria

*equal contribution

**Supplementary Material and Methods**

**Skin biopsy preparation**

Written informed consent was obtained from tissue donors and the study was approved by the Ethics Committee of the Medical Faculty, University of Würzburg (96/11). Human dermal fibroblasts were isolated from healthy individuals and cultured in fibroblast growth medium containing Dulbecco's modified eagle's medium (DMEM; PAN Biotech, Aidenbach, Germany), 10% fetal bovine serum (FBS; Gibco, ‎Carlsbad, California), and 1 µg/ml Gentamicin (Thermo Fisher Scientific, Waltham, Massachusetts, USA) at 37°C in 5% CO_2_.

**Reprogramming of human fibroblasts into hiPSCs**

For the generation of the hiPSC line UKWMPi001-B fibroblasts of a healthy donor were reprogrammed using the CytoTune-iPS Reprogramming Kit 2.0 (Thermo Fisher Scientific, Waltham, MA, USA) as previously described (Jansch et al 2018). 75x10^3^ fibroblasts per well of a 12-well plate were transduced with Sendai virus for 24 h. After 7 days cells were seeded on irradiated mouse embryonic fibroblasts (MEF; 4x10^5^/9.6 cm^2^) (Amsbio, Abingdon, U.K.) in reprogramming medium (KnockOut™ DMEM/F12 (Gibco), 20% KnockOut™ Serum Replacement (Gibco), 1 mM L-glutamine (Gibco), 1% MEM non-essential amino acid solution (100x, NEAA; PAN Biotech), 100 μM β-mercaptoethanol (Gibco), 10 ng/mL human fibroblast growth factor 2 (FGF2; Gibco) and 200 μM ascorbic acid (Sigma-Aldrich, St. Louis, Missouri, USA). Colonies with iPSC-like appearance were manually transferred on day 26 after infection to Matrigel (Corning, New York, USA)-coated 48-well plates (Greiner, Bio-one, Kremsmünster, Austria) in StemMACS™ iPS-Brew XF (Miltenyi Biotech, Bergisch Gladbach, Germany). For long-term culturing hiPSCs were kept in StemMACS™ iPS-Brew XF in a 6-well format with daily medium changes. Cells were split every three to four days with addition of 10 µM ROCK inhibitor (Miltenyi Biotech) for 24 hours after Accutase (Merck Millipore, Darmstadt, Germany) treatment and frozen using KnockOut™ Serum Replacement supplemented with 10% DMSO (Sigma-Aldrich).

**RNA Extraction and quantitative real-time polymerase chain reaction (qRT-PCR)**

Total RNA was isolated from 3x10^6^ iPSCs as well as neurons (RNeasy mini Kit; Qiagen, Hilden, Germany). 500 ng RNA was reverse transcribed with QuantiTect Reverse Transcription Kit (Qiagen). The iQ™ SYBR® Green Supermix (BioRad, Munich, Germany) was used to study the mRNA expression levels of *TPH2* (forward 5´to 3´: ACGGAGAGGGTTTTCCCTG; reverse 5´to 3´: TGCCAAGTAGCTGATGCTCT), *glutamate decarboxylase 1* (*GAD1*) (forward: CAAACATTTATCAACATGCGCTTC; reverse: CTATGACACTGGAGACAAGGC), *tyrosine hydroxylase* (*TH*) (forward: CCTGGTTCCCAAGAAAAGTGTCAGA; reverse: CCCTTCAGCGTGGTGTAGACCTC), and the pluripotency markers *NANOG* (forward: CTGAGATGCCTCACACGGAG; reverse: TGTTTGCCTTTGGGACTGGT), *OCT3* (forward: CCCACACTGCAGCAGATCA; reverse: TGTGCATAGTCGCTGCTTGA), and *REX1* (forward: AGGTGGCATTGGAAATAGCAGA; reverse: AGTGGGGTGGGTTTGCCTA). Components were applied according to manufactured protocol. For all conducted qPCRs, samples were tested in triplicates using 384-well plates. All PCRs were run in the thermal cycler CFX384 controlled by the software CFX manager 3.0. QuantiTect Primer Assays (Qiagen) of the genes *glyceraldehyde-3 phosphate dehydrogenase* (*GAPDH*) (Hs_GAPDH_1_SG QuantiTect Primer Assay), *beta-actin* (*ACTB*) (Hs_ACTB_1_SG QuantiTect Primer Assay (QT00095431)) and *polyubiquitin-C* (*UBC*) (Hs_UBC_1_SG QuantiTect Primer Assay) were used as reference genes in order to normalize the data for *TPH2*, *GAD1*, and *TH* expression levels. Relative expression levels of *NANOG*, *OCT3*, and *REX1* were normalized by the expression of the housekeeping genes *PSMB4* (forward: GGACATGCTTGGTGTAGCCT, reverse: AGCGTTCTACTAAGTCGCGG) and *RS27A* (forward: GGTTAAGCTGGCTGTCCTGAA, reverse: AGAAGGGCACTCTCGACGAA). Data analysis as well as data processing was done using the programs CFX-Manager 3.0 and LinReg.

**Immunofluorescence analysis**

Cells were washed with phosphate-buffered saline (PBS; Thermo Fisher), pre-fixed using a 1:1 dilution of NMM and 4% paraformaldehyde (Roth, Karlsruhe, Germany) for 5 min and finally fixed using 4% paraformaldehyde for 15 min at room temperature (RT). Cells were blocked (10% FBS, 0.1 % BSA in PBS) and permeabilized with 0.2% Triton X-100 (Sigma-Aldrich) for 45 min at RT. For specific visualization of 5-HTT, no permeabilization was performed and cells were blocked using 10% FBS and 0.1 % BSA in PBS. Primary antibodies were applied and incubated overnight at 4°C. The next day, cells were washed with PBS and incubated with secondary antibodies (1 h, RT) together with DAPI (4',6-diamidino-2-phenylindole, Sigma-Aldrich; 300 nM). All antibodies, sources, dilutions and usage for specific methods are listed in Supplementary Table 1 and Supplementary Table 2.

Additionally, the visualization of the exact CDH13 expression along the cell required a life cell staining. Therefore, living iPSC-derived neurons were first incubated with the primary antibody against CDH13 for 1 h on ice and finally fixed and incubated with further primary antibodies overnight at 4°C. Following steps were performed as described above.

**Evaluation of differentiation efficiency**

To determine the efficiency of 5-HT specific neuron differentiation, the ratio of TPH2+, TH+ and GABA+ cells to ß-TubulinIII positive (ßTubIII+) cells was calculated. To this end, the mature neurons were stained with appropriate markers according to methods described in section 2.5 and five areas per independent experiment (n=3) were randomly selected.

**3D reconstruction**

Movies illustrating 3D reconstruction were done using Imaris (Bitplane, Belfast, U.K.). The images used were taken with SIM at intervals of 125 nm. The fluorescence images were represented by surface visualization and the animation was conducted by rotation and zooming. The final product was exported as an .avi file.

**Supplementary Legends**

**Supplementary Video 1.** 3D reconstruction of double IF of TPH2 and CDH13. Movies illustrating 3D reconstruction using Imaris. TPH2+ neurons are labeled in green (Alexa Fluor 555) and CDH13 immunofluorescence is represented in red (Alexa Fluor 647).

**Supplementary Table 1.**

List of primary antibodies used in this study.

| **Name** | **Company** | **Catalog#** | **Host** | **Dilution** | **Usage** |
| --- | --- | --- | --- | --- | --- |
| SOX1 Antibody | R&D Systems | AF3369 | Goat | 1:150 | Epifluorescence |
| HNF-3β Antibody (H-4) | Santa Cruz | sc-374376 | Mouse | 1:50 | Epifluorescence |
| Purified anti-Pax-6 Antibody | Biolegend | 901301 | Rabbit | 1:100 | Epifluorescence |
| Human Nestin Antibody | R&D Systems | MAB1259 | Mouse | 1:100 | Epifluorescence |
| Human/Mouse/Rat SOX2 Antibody | R&D Systems | MAB2018 | Mouse | 1:500 | Epifluorescence |
| NKX2.2 | DSHB Hybridoma Bank | 74.5A5 | Mouse | 1:50 | Epifluorescence |
| Anti-NKX6-1 Antibody | Sigma | HPA036774 | Rabbit | 1:500 | Epifluorescence |
| Anti-GATA2 Antibody | Sigma | HPA005633 | Rabbit | 1:500 | Epifluorescence |
| Anti-HOXA2 Antibody | Abcam | Ab229960 | Rabbit | 1:500 | Epifluorescence |
| Pax-3/7 Antibody (B-5) | Santa Cruz | sc-365843 | Mouse | 1:50 | Epifluorescence |
| Anti-βIII Tubulin mAb | Promega | G7121 | Mouse | 1:1000 | Epifluorescence;  SIM |
| Glial Fibrillary Acidic Protein (GFAP) | Dako | Z0344 | Rabbit | 1:5000 | Epifluorescence |
| Tryptophan hydroxylase 2 Antibody (TPH2) | Novus Biologicals | NB100-74555 | Rabbit | 1:2000 | Confocal;  SIM |
| Tryptophan hydroxylase 2 Antibody (TPH2) | Novus | NB100-2447 | Goat | 1:100 | Confocal |
| 5-HT (Serotonin) Antibody | Immunostar | 20080 | Rabbit | 1:500 | Confocal |
| 5-HT (Serotonin) Transporter Antibody | ImmunoStar | 24330 | Rabbit | 1:5000 | Confocal |
| Anti-GABA Antibody | Sigma-Aldrich | A2052 | Rabbit | 1:1000 | Confocal |
| Anti-Tyrosine Hydroxylase Antibody (TH) | abcam | ab112 | Rabbit | 1:1000 | Confocal |
| MAP2 | Synaptic Systems | 188 004 | Guinea pig | 1:1000 | Epifluorescence  Confocal |
| Tau | Synaptic systems | 314004 | Guinea pig | 1:500 | Epifluorescence |
| Anti-NeuN Antibody, clone A60 | Merck Millipore | MAB377 | Mouse | 1:100 | Epifluorescence |
| Human Cadherin-13 Antibody (CDH13) | R&D Systems | AF3264 | Goat | 1:200 | SIM |
| Bassoon | Synaptic Systems | 114004 | Guinea pig | 1:300 | *d*STORM  Confocal |
| Homer | Synaptic Systems | 160 011 | Mouse | 1:300 | *d*STORM |
| Anti-PSD95 Antibody, clone K28/43 | Merck Millipore | MABN68 | Mouse | 1:500 | Confocal |

**Supplementary Table 2.**

List of secondary antibodies used in this study.

| **Name** | **Company** | **Catalog#** | **Specifity** | **Dilution** | **Usage** |
| --- | --- | --- | --- | --- | --- |
| Donkey anti-Mouse IgG (H+L) ReadyProbes™ Secondary Antibody, Alexa Fluor 488 | Thermo Fisher | R37114 | IgG (H&L) | 1:400 | Epifluorescence;  Confocal;  SIM |
| Donkey anti-Rabbit IgG (H+L) Highly Cross-Adsorbed Secondary Antibody, Alexa Fluor 488 | Thermo Fisher | A21206 | IgG (H&L) | 1:400 | Epifluorescence |
| Goat anti-Guinea Pig IgG (H+L) Highly Cross-Adsorbed Secondary Antibody, Alexa Fluor 488 | Thermo Fisher | A-11073 | IgG (H&L) | 1:400 | Epifluorescence;  Confocal |
| Donkey anti-Rabbit IgG (H+L) Highly Cross-Adsorbed Secondary Antibody, Alexa Fluor 555 | Thermo Fisher | A31572 | IgG (H&L) | 1:400 | Epifluorescence;  Confocal;  SIM |
| Donkey anti-Goat IgG (H+L) Cross-Adsorbed Secondary Antibody, Alexa Fluor 555 | Thermo Fisher | A21432 | IgG (H&L) | 1:400 | Epifluorescence |
| Donkey anti-Mouse IgG (H+L) Highly Cross-Adsorbed Secondary Antibody, Alexa Fluor Plus 555 | Thermo Fisher | A32773 | IgG (H&L) | 1:400 | Epifluorescence |
| Goat anti-Mouse IgG (H+L) Cross-Adsorbed Secondary Antibody, Alexa Fluor 532 | Thermo Fisher | A-11002 | IgG (H&L) | 1:400 | *d*STORM |
| Goat anti-Rabbit IgG (H+L) Cross-Adsorbed Secondary Antibody, Alexa Fluor 532 | Thermo Fisher | A-11009 | IgG (H&L) | 1:400 | *d*STORM |
| Donkey anti-Goat IgG (H+L) Cross-Adsorbed Secondary Antibody, Alexa Fluor 647 | Thermo Fisher | A-21447 | IgG (H&L) | 1:400 | SIM;  *d*STORM |
| Alexa Fluor® 647 AffiniPure Donkey Anti-Guinea Pig IgG (H+L) | Jackson ImmunoResearch | IR706-605-148 | IgG (H&L) | 1:400 | *d*STORM |
