# Supplementary figures and images for: Serotonin-specific neurons differentiated from human iPSCs form distinct subtypes with synaptic protein assembly
Source: J Neural Transm (Vienna). 2021 Feb 9;128(2):225–41. doi: 10.1007/s00702-021-02303-5 (PMC7914246; doi:10.1007/s00702-021-02303-5)

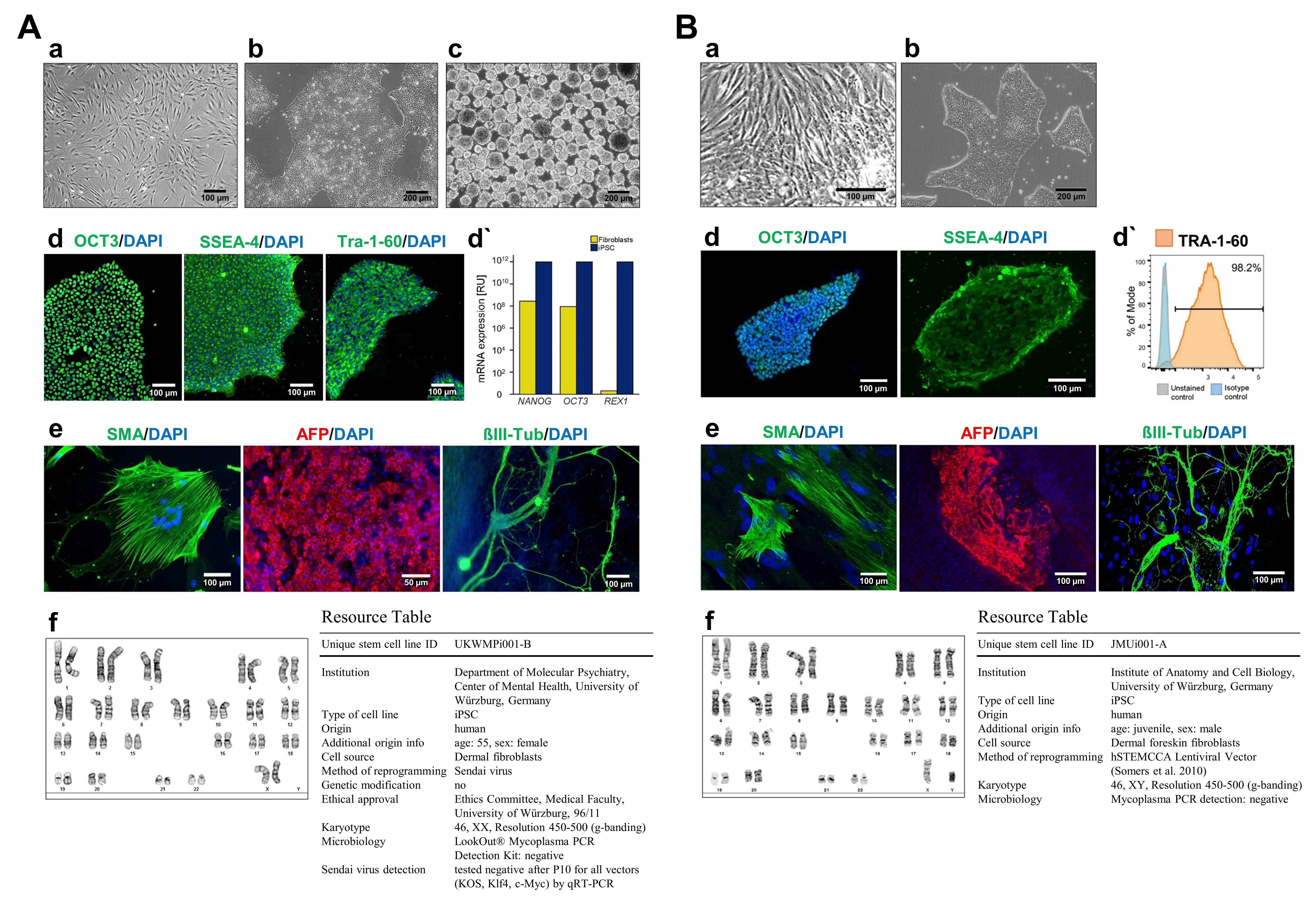

Supplement: Supplementary file 3 — Supplementary file3 Supplementary Fig. S1. Characterization of hiPSC lines. hiPSC lines (A: UKWMPi001-B, B: JMUi001-A) used in this study were gained by reprogramming of fibroblasts (a), showed the typical ES-like morphology in adherent culture (b), and built EBs in suspension culture (c) (only shown for line UKWMPi001-B). Both cell lines expressed pluripotency markers as assessed by immunostaining (d) and quantitative measurements as demostrated by qRT-PCR (A, d`) or FACS analysis (B, d`). EBs spontaneously differentiated into cells of the 3 germ layers (e). Chromosomal integrity was proven by karyotyping (f). Further description of the cell lines is given in the resource tables (JPG 3246 KB) [file 702_2021_2303_MOESM3_ESM.jpg]

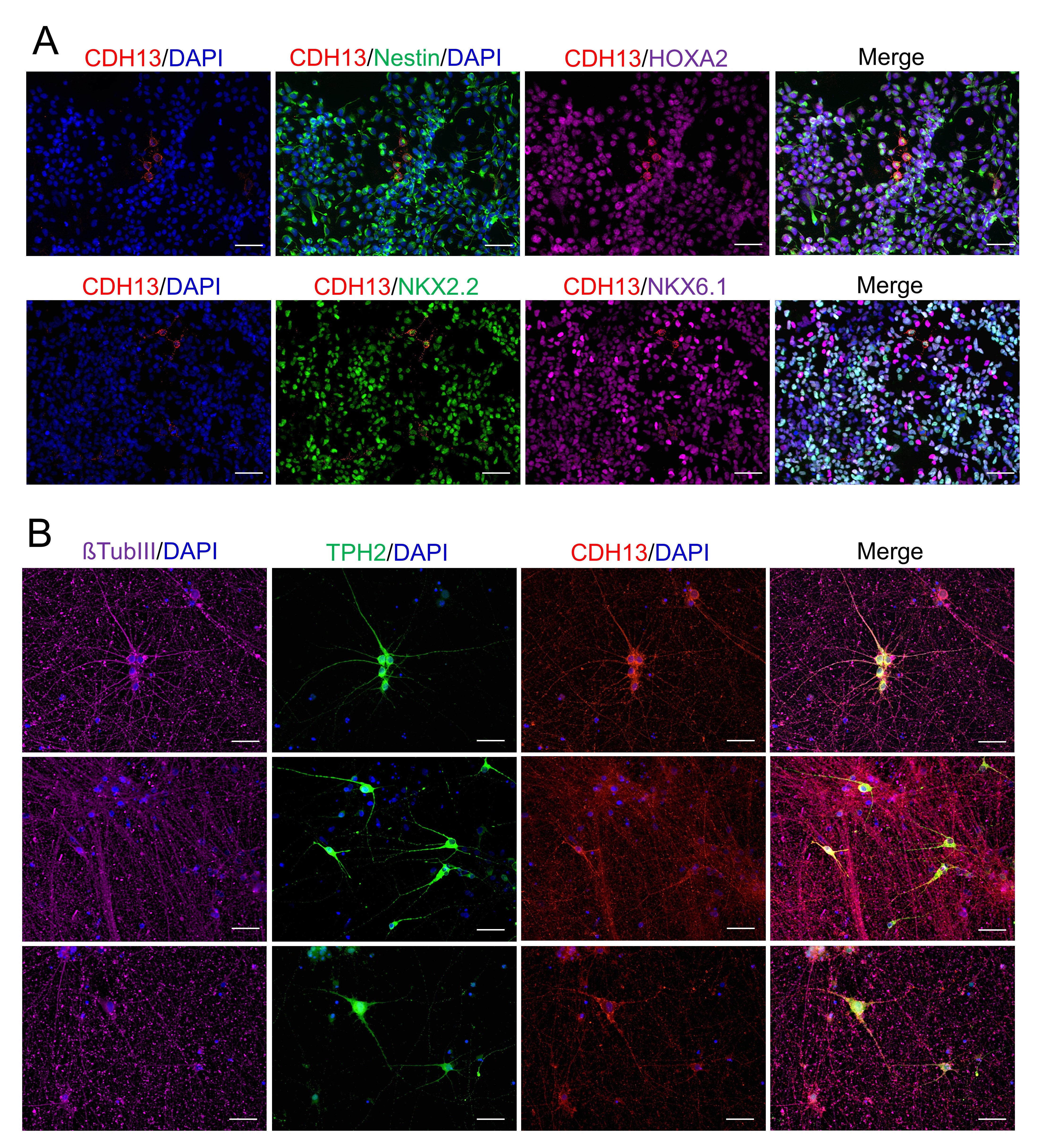

Supplement: Supplementary file 4 — Supplementary file5 Supplementary Fig. S2. CDH13 expression in 5-HT specific NPCs (A) and neurons (B). Representative epifluorescence imaging of CDH13 expression in NPCs (A) shows that CDH13 is expressed in only few 5-HT specific NPCs. The serotonergic fate of these NPCs is depicted by expression of the general NPC marker Nestin together with the rostral hindbrain marker HOXA2 as well as the ventral rostral hindbrain markers NKX2.2 and NKX6.1. After 8 weeks of neuronal maturation CDH13 immunoreactivity is spread equally across the whole culture with about 40% of TPH2+ and ßTUB3+ cells showing immunoreactivity for CDH13 as depicted in representative images (B). Scale bar: 50 μm (JPG 4056 KB) [file 702_2021_2303_MOESM4_ESM.jpg]
